# Yeast Additive Effects on Dry Matter Intake, Milk Production, Milk Composition, and Ruminal Metabolism in Lactating Dairy Cattle †

**DOI:** 10.3390/ani16131970

**Published:** 2026-06-25

**Authors:** Michaela R. Plowman, Barry D. Lambert, James P. Muir, Walter F. Owsley, Kimberly B. Wellmann

**Affiliations:** 1 Department of Animal Science, Tarleton State University, Stephenville, TX 76402, USA; mplowman@tarleton.edu (M.R.P.);; 2 Department of Wildlife & Natural Resources, Tarleton State University, Stephenville, TX 76402, USA; 3 Texas A&M AgriLife Research, Stephenville, TX 76401, USA

**Keywords:** dairy, milk production, rumen pH, yeast

## Abstract

*Saccharomyces cerevisiae* yeast products are used to stabilize dairy cattle rumen environments. In this study, multiparous lactating Holstein–Jersey cross cows in late lactation (*n* = 12; ±150 DIM) were used in a replicated 4 × 4 Latin square design over four 21 d periods to evaluate yeast supplementation on dry matter intake (DMI), milk production, milk components, rumen pH, and redox potential (E_h_). A subset of four ruminally cannulated cows were selected for rumen measurements. Treatments included no additive (CON), 14 g yeast culture additive (YCA), 5 g active dry yeast (ADY), and 5 g ADY + 5 g yeast extract additive (YEA) top-dressed once daily. Data were analyzed using GLIMMIX in SAS 9.4, with significance at *p* ≤ 0.05 and tendencies at *p* ≤ 0.15. Dry matter intake did not differ among treatments (*p* > 0.15) but decreased over time (*p* < 0.05). Milk production, efficiency, lactose, and total solids were not affected (*p* > 0.15). YCA tended to increase milk fat and fatty acid fractions compared with CON (*p* < 0.15). Protein in milk was greater in CON (*p* < 0.05; *p* < 0.05) than YCA and YEA. Active dry yeast treatments increased the frequency of a buffered rumen (*p* < 0.05) and promoted a more reducing ruminal environment, characterized by lower redox potential and conditions favorable for anaerobic fermentation (*p* < 0.05). Overall, supplementing active dry yeasts to dairy cows in late lactation did not affect DMI, production efficiency, or milk quality, but may maintain rumen pH stability.

## Simple Summary

Yeast products, commonly derived from strains of *Saccharomyces cerevisiae*, are used worldwide to help stabilize the anaerobic rumen environment in dairy cattle. This study evaluated the effects of commercially available yeast products on dry matter intake, milk production, milk components, and ruminal conditions in lactating Holstein–Jersey cows. Overall, yeast supplementation supported milk component responses while cows maintained milk production under a high-concentrate dietary challenge and declining dry matter intake. These findings suggest that yeast products support productive performance in lactating dairy cows while aiding in the maintenance of a stable rumen environment.

## 1. Introduction

Dairy cattle are often managed under intensive production practices to maximize milk yield and milk quality [1]. To support high levels of production, dairy rations commonly contain large amounts of soluble carbohydrates to maintain a positive energy balance during peak lactation [1]. However, high soluble carbohydrate intake has been associated with an increased risk of metabolic disturbances in the rumen, including subacute ruminal acidosis (SARA) [2]. Common signs of SARA include decreased dry matter intake (DMI), altered rumen fermentation, and reduced overall animal performance [3]. Acids produced during the fermentation of grains and forages contained in a balanced total mixed ration (TMR) generally keep rumen pH slightly below neutral (pH = 7.0) [4]. Sub-acute ruminal acidosis occurs when the pH of the rumen declines below 5.8 for a prolonged period, normally ranging from 3–5 h [5]. Decreased DMI related to SARA can be attributed to several factors: lack of rumen buffering caused by heat stress, increased respiratory rate, respiratory alkalosis, and low blood bicarbonate concentrations [5]. The resulting SARA in the summer months could include atypical meal patterns in response to heat avoidance and ration formulation errors made when nutritionists attempt to compensate for reduced DMI during heat stress by decreasing dietary fiber [6].

Yeast, classified as a direct-fed microbial (DFM), works to enhance production performance, alter ruminal fermentation, or improve nutrient utilization [7]. Yeast products are used worldwide to improve feed and fiber digestibility, support ruminal fermentation, and enhance milk production, with *Saccharomyces cerevisiae* being one of the most common yeast species in dairy cattle diets [8]. There is ongoing debate on the effectiveness of the type of yeast product used, with discussion centered on active dry yeast products, yeast culture additive, and yeast cell wall products. Active dry yeast is the most common inclusion in dairy diets, often a granular substance and distinct strain of *S. cerevisiae* [9]. Yeast culture additives contain both yeast and fermentation metabolites resulting from the fermentation process to produce the product [10]. Yeast cell wall products include both intracellular components and yeast cell wall fragments, derived from lysed yeast cells using acids, enzymes, or high-salt solutions [11]. The yeast cell wall products contain beta-glucans mannan-oligosaccharides which may help to mitigate pathogen colonization in an animal’s intestinal tract by aiding immune response and inhibiting the growth of Gram-negative bacteria, providing potential prebiotic and immunomodulatory effects [11]. If rumen pH needs alteration or stabilization, *S. cerevisiae* products increase ruminal pH, leading to fewer events of acidosis driven by high-cereal grain diets in dairy cows and allowing for a more conducive ruminal environment without milk fat depression by the overproduction of volatile fatty acids (VFA) [12].

Milk yield and milk fatty acids (FA) are essential factors in analyzing yeast product effectiveness and the herd production level. Milk yield and milk fatty acid profiles are important response variables for evaluating the effectiveness of yeast products, as yeast supplementation has been shown to influence intake, rumen fermentation, and milk production, while milk fatty acid composition reflects dietary and ruminal fermentation changes in dairy cattle [13,14]. *S. cerevisiae* products may influence the rumen environment by altering volatile fatty acid profiles, supporting ruminal pH, and promoting fermentation patterns that favor energy-yielding end products under high-concentrate dietary conditions [12]. However, changes in acetate production alone should not be interpreted as direct evidence of reduced methane production, as acetate formation is generally associated with hydrogen production and may contribute to methanogenesis depending on ruminal conditions [12]. Yeast supplementation may influence methane-related fermentation dynamics by altering volatile fatty acid profiles and ruminal fermentation patterns [15]. Predicting the methane yield from dairy cows offers a simple solution for determining if the rumen is losing energy by methane production and offers an insight to the possible mitigation of methane production.

Souza et al. [16] illustrated the positive relationship between the rumen environment using ruminal pH measurements and milk fat, stating that increasing rumen pH increases fiber digestibility and acetate supply for milk production. The positive relationship between rumen efficiency, as indicated by rumen pH and milk fat, shows increasing fiber digestibility and acetate production. As a result, VFA production can be used to estimate energy losses.

Measuring rumen pH and redox potential (E_h_) can give an indication of microbiological activity and dynamics of fermentation [17]. Redox conditions in the digestive tract of animals determine whether aerobic oxidation or anaerobic fermentation of nutrients should prevail and have major impacts on the digestion, metabolism, and assimilation of ingested nutrients [18]. Under normal rumen conditions, E_h_ is negative, indicating the absence of oxygen and strong reducing power of the ruminal contents. Quantifiable measures of pH and E_h_ will increase the reliability of predicted values for milk fat (MF) and VFAs representing energy loss or gain within the animal.

Although yeast products are commonly supplemented in dairy cattle diets to support ruminal fermentation and production performance, responses to supplementation remain variable and may depend on yeast product type, strain, dose, diet composition, and stage of lactation [7,8]. Previous studies have evaluated yeast supplementation effects on dry matter intake, ruminal pH, volatile fatty acid concentration, milk yield, milk components, and methane production; however, these responses are often evaluated independently or within a single yeast product category [12,13,14]. Ruminal pH is frequently used as an indicator of rumen stability indicating acid-base status [19]. Ruminal pH measurement alone does not fully describe the anaerobic and reducing conditions that support microbial fermentation [19]. Redox potential provides additional insight into the reducing capacity of ruminal contents and may better reflect changes in the anaerobic rumen environment associated with microbial activity and fermentation dynamics [15,16,17,18].

A gap remains in understanding how different yeast product types, including active dry yeast, yeast culture additives, and yeast cell wall products, comparatively and simultaneously influence ruminal fermentation and production responses when evaluated within the same experimental framework. Specifically, limited research has evaluated these products using combined measures of ruminal pH, redox potential, volatile fatty acid profiles, predicted methane production, milk yield, and milk composition. Redox potential included as a primary outcome reflects the reducing conditions of the rumen. Measuring E_h_ alongside pH provides a more complete assessment of rumen stability by evaluating both acidity and the anaerobic environment that supports microbial fermentation. This may be particularly relevant when evaluating yeast products, whose effects may extend beyond changes in ruminal pH alone. This approach provides a more comprehensive evaluation of yeast product effectiveness by linking rumen fermentation conditions with animal performance outcomes. Therefore, this study was designed to evaluate the effects of different yeast products on dry matter intake, milk production, milk components, milk fatty acid profiles, predicted methane production, and rumen environmental parameters, including ruminal pH and redox potential, in lactating dairy cattle.

## 2. Materials and Methods

All procedures were approved by the Tarleton State University Animal Care and Use Committee (Protocol 10-017-2021). Multiparous lactating, Holstein–Jersey cross cattle in late lactation [*n* = 12; ±150 days in milk (DIM)] were used in a replicated 4 × 4 Latin square design consisting of four 21 d periods to evaluate the effect of yeast supplementation on DMI, milk production, and milk components. Each period was 21 d in length, with the first 16 d used as an adaptation and milk sample collections on d 18–21. Blinding of personnel during data collection was not feasible due to the diet-based treatment structure and management requirements of the replicated Latin square design; however, potential bias was minimized through objective outcome measures and consistent data collection procedures across all cows, treatments, and periods.

A subset of the sample population (*n* = 4; 150 ± 30 DIM) were selected to be ruminally fistulated and cannulated based on the available space in the paralumbar fossa, to evaluate the effect of yeast on rumen pH and E_h_. Each period was 21 d in length with the first 16 d used as an adaptation, the d 17 used for rumen fluid sample collection, and the d 18–21 for milk sample collection. Cows entered a backgrounding period on d -16 to allow adaptation to the basal diet and feeding procedures. On d -16, cattle were ruminally fistulated and cannulated and allowed surgical recovery through d 0.

Cattle were housed in a single pen at the Southwest Regional Dairy Center (Stephenville, TX, USA) and randomly assigned to Calan gates for feeding to facilitate treatment delivery and intake measurement. A basal diet formulated to meet or exceed NASEM [20] recommendations for lactating dairy cows was fed twice daily (0630 and 1600 h; 2/3 and 1/3 of daily feed delivery, respectively) as a total mixed ration to allow for approximately 5% feed refusals. Cows were milked twice daily (0600 and 1800 CST).

Cows were selected for similar daily production based on previous 305 d milk production and number of days dry. Treatments were randomly assigned within replicates, with treatment dosage determined by directions from the product manufacturer. Treatments consisted of 1 of the 4 treatment diets with all treatments top-dressed with the morning feed delivery to ensure treatment consumption.

The basal diet was formulated to meet or exceed NASEM [20] recommendations for mid to late lactating dairy cows, similar to other dairy rations fed in Erath County, TX, USA (Table 1), which contained ≥26% and ≤30% starch.

### 2.1. Treatments

- Basal diet—no yeast culture or yeast probiotics (CON);
- Basal diet + 14 g/day yeast culture additive (commercially available proprietary strain of *S. cerevisiae* yeast fermentate/post-biotic) (YCA);
- Basal diet + 5 g/day active dry yeast (commercially available *S. cerevisiae* CNCM I-4407) (mean count 1.0 × 10^10^ CFU/g) (ADY);
- Basal diet + 5 g/day active dry yeast active dry yeast (commercially available *S. cerevisiae* CNCM I-4407) (mean count 1.0 × 10^10^ CFU/g) + 5 g/day yeast extract additive (commercially available primary culture and purification of proprietary blend of *S. cerevisiae* yeast post-biotic) (YEA).

### 2.2. Feed and Refusal Analysis

Daily feed samples were collected at delivery of basal diet, then composited into 1 feed sample/wk (12 samples total). Feed refusals were sampled daily and pooled by week for each animal for analysis (144 samples total). All feed and refusal samples were sent to Dairy One (Ithaca, NY, USA) for laboratory nutritive value analysis. Samples were analyzed by near-infrared reflectance spectroscopy using Dairy One Forage Laboratory NIRS calibrations. All treatments were stored in a climate-controlled room (~21 °C).

### 2.3. Production Analysis

Daily individual DMI was calculated based on daily feed delivery, daily feed refusals (collected at 05:30 h), and the DM percent of each of those fractions. Daily milk production and weekly milk component data were collected at 06:00 and 18:00 h. Milk production efficiency was calculated thereafter.

### 2.4. Milk Component Analysis

Milk samples were collected on d 18–21 of each of the four periods, preserved with broad spectrum microtabs (Broad Spectrum Microtabs II™, Advanced Instruments, Norwood, MA, USA), stored at 1–4 °C, and delivered to the ADM laboratory (Clovis, NM, USA) for somatic cell count (SCC), milk fat (MF), protein, lactose, solids-not-fat (SNF), total solids (TS), milk urea nitrogen (MUN), *de novo* FA, mixed FA, preformed FA, mean chain length, mono-unsaturated fatty acid (MUFA), and non-esterified fatty acid (NEFA) content determination. Milk fatty acid profiles were analyzed at the Animal and Food Sciences Laboratory (Texas Tech University, Lubbock, TX, USA). Fatty acids methyl ester (FAME) were prepared following the methylation technique by O’Fallon et al. [21]. Following the methylation, one gram of the homogenate was put into a glass vial, and an internal standard was added (tridecanoic acid, 0.5 mg/mL in methanol). Vials were then incubated at 55 °C in a water bath, and FAME were extracted using Hexane prior to analysis. Hexane extracts were analyzed by an Agilent 7890A gas chromatographer (Agilent Scientific Instruments, Santa Clara, CA, USA).

Milk FA profiles were also determined as a means of methane production estimation using the following equation from Dijkstra et al. [15]:

CH_4_ (g/kg DM) = 24.6 ± 1.28 + 8.74 ± 3.581 × C17:0 *anteiso* FA − 1.97 ± 0.432 × *trans*-10 + 11 C18:1 − 9.09 ± 1.444 × *cis*-11 C18:1 + 5.07 ± 1.937 × *cis*-13 C18:1 (1)

(individual FA in g/100 g FA; R^2^ = 0.73 after correction for experiment effect).

### 2.5. Ruminal Measurement Analysis

Ruminal pH and E_h_ were recorded one time/min for 24 h on d 16 of each period of the Latin square beginning at 0530 h in each of the 4 cannulated cows resulting in 1440 observations/day/period for each cow. Rumen fluid was collected for validation of E_h_ and volatile fatty acid (acetate, propionate and butyrate) analysis at −1, 1, 2, 3, and 4 h respective to the 06:30 h feeding on d 17 of each period. Pre-prandial samples collected at h 1 were used to evaluate baseline VFA concentrations, whereas postprandial samples collected at 1, 2, 3, and 4 h after feeding were pooled prior to VFA analysis. Rumen samples were analyzed for VFA content at the Animal and Food Sciences Laboratory (Texas Tech University, Lubbock, TX, USA). Rumen fluid was centrifuged and 1 mL of supernatant was removed from the top and placed into a microcentrifuge tube. A total of 100 µL of phosphoric acid was added to the sample. 0.3000 ± 0.0050 g NaCl was added into a GC vial, followed by 100 µL of phosphorated sample and 900 µL of milliQ water to the GC vial. The mixture was thoroughly vortexed. Samples were analyzed by an Agilent 7890A gas chromatographer (Agilent Scientific Instruments, Santa Clara, CA, USA).

### 2.6. Animal Exclusion

Animals were evaluated for inclusion prior to study initiation based on health status, physiological condition, and suitability for the experimental design. Animals were excluded a priori if they displayed clinical signs of illness, lameness, injury, abnormal behavior, or any condition that could compromise animal welfare or interfere with treatment responses or data collection. Additional exclusion criteria included failure to meet study-specific requirements, such as age, body weight, production stage, parity, pregnancy status, or prior treatment history, as applicable. Animals requiring medical intervention prior to the start of the trial or deemed unsuitable by research personnel or the attending veterinarian were not enrolled in the study.

If cows became sick, lame, or otherwise required medical attention during the trial, they were removed from data collection for an appropriate recovery period to ensure animal welfare and to prevent illness or treatment effects from confounding study outcomes. Return to the study was based on recovery status and approval by research personnel and/or the attending veterinarian.

During the last period, one cow developed mastitis and was excluded from data collection for that period. Data collected from that cow during previous periods were not identified as outliers; therefore, those data were retained in the analysis. The cow was included in the study until diagnosis and subsequent treatment in the hospital pen during Period 4.

### 2.7. Statistical Analysis

All data were analyzed using the GLIMMIX procedure of SAS 9.4 (SAS Institute Inc., Cary, NC, USA). Weekly milk components, ruminal volatile fatty acids, and milk fatty acid data were analyzed as a replicated 4 × 4 Latin square. The model included square, cow nested within square, period nested within square, and treatment. Dietary treatment was considered a fixed effect, whereas square, cow nested within square, and period nested within square were considered random effects. The model was:

Yijkl = μ + Si + Cj(Si) + Pk(Si) + Tl + eijkl

where Yijkl is the dependent variable, μ is the overall mean, Si is the random effect of square, Cj(Si) is the random effect of cow nested within square, Pk(Si) is the random effect of period nested within square, Tl is the fixed effect of treatment, and eijkl is the residual error.

Dry matter intake, milk production, efficiency, and other ruminal data were analyzed as split-plot repeated-measures data over time. The whole-plot model included square, cow nested within square, period nested within square, and treatment. The sub-plot model included time and the treatment × time interaction. The model was:

Yijklm = μ + Si + Cj(Si) + Pk(Si) + Tl + Mm + Tl × Mm + eijklm

where Yijklm is the dependent variable, μ is the overall mean, Si is the random effect of square, Cj(Si) is the random effect of cow nested within square, Pk(Si) is the random effect of period nested within square, Tl is the fixed effect of treatment, Mm is the fixed effect of time, Tl × Mm is the fixed interaction of treatment and time, and eijklm is the residual error. Treatment effects were tested using the whole-plot error term of cow × period nested within square × treatment. Time and treatment × time effects were tested using the sub-plot residual error. Repeated measures over time were modeled within cow using an autoregressive covariance structure [AR(1)].

Values from continuous rumen data were averaged by 10 min intervals for visualization and general linear model analysis. Ruminal volatile fatty acids were analyzed using the replicated 4 × 4 Latin square model described above, with square, cow nested within square, period nested within square, and treatment included in the model. Rumen pH and E_h_, which were measured every 1 min, were converted from continuous variables to binary categorical variables. Data were transformed using a pH threshold of 6.0, with observations classified as SARA when pH < 6.0 and buffered when pH ≥ 6.0. Redox potential was categorized using an E_h_ threshold of −190 mV, with observations classified as reducing when E_h_ ≤ −190 mV and oxidizing when E_h_ > −190 mV. Binary categorical rumen pH and E_h_ data were analyzed using chi-squared tests of independence to evaluate associations among the four treatments and the two categories within rumen pH and E_h_, respectively. Due to the limited number of ruminally cannulated cows, ruminal measurements should be interpreted with consideration of reduced statistical power and the potential for Type II error. Thus, biologically relevant ruminal responses may not have been detected as statistically significant. Repeated-measures analyses were used when appropriate to account for within-animal correlations over time and improve precision. In addition, tendencies were discussed at 0.05 < *p* ≤ 0.15 to support interpretation of biologically meaningful responses that may warrant further evaluation in studies with a larger number of experimental units. An α level of 0.05 was used to determine significance, with tendencies discussed at 0.05 < *p* ≤ 0.15 for both production measures and ruminal measures.

## 3. Results

### 3.1. Production Efficiency

DMI did not differ (*p* = 0.40; Table 2) among treatments throughout the trial but did decrease with each subsequent period over time (*p* < 0.01). Upon the conclusion of the adaptation period for each treatment, no differences (*p* = 0.88) were observed for fat-corrected milk (FCM) yield between treatments. Production efficiency (FCM/DMI) also did not differ (*p* = 0.84) across treatments. For predicted methane production (g of CH_4_/kg DMI) using an equation developed by Dijkstra et al. [15], treatments did not differ (*p* = 0.52).

### 3.2. Milk Components

Milk SCC, lactose, TS, MUN, MCL, MUFA, and NEFA did not differ (*p* > 0.15) among treatments (Table 3). Milk SNF tended to increase (*p* = 0.12) in CON compared to ADY and YEA with YCA not different from any of the treatments. Milk fat (*p* = 0.10), *de novo* FA/g as measured in the milk (*p* = 0.12), mixed FA/g as measured in the milk (*p* = 0.11), and preformed FA/g as measured in the milk (*p* = 0.13) tended to be greater for YCA compared to CON, but ADY and YEA were not different (*p* > 0.15) than any of the treatments. On a basis relative to total FA, *de novo*, mixed, and preformed FA did not differ (*p* > 0.15) between treatments. Crude protein was greater (*p* = 0.01) in CON compared to YCA and YEA, and ADY was not different (*p* > 0.15) than any of the treatments.

### 3.3. VFA Concentrations

Pre- and post-prandial VFA profiles showed no treatment by sampling time interaction; therefore, main effects of sampling time and treatment were investigated (Table 4). Pre- and post-prandial concentrations were only different for isobutyrate, where pre-prandial concentrations were greater (*p* = 0.05) than post-prandial concentrations. Acetate production for CON decreased (*p* = 0.02) compared to yeast treatments. Propionate concentrations were greater (*p* = 0.03) for CON compared to YCA and ADY, while YEA maintained intermediate concentrations. The acetate:propionate ratio was greater (*p* = 0.03) in YCA than CON; ADY and YEA were intermediate and not different from CON or YCA. Butyrate concentrations were greater (*p* = 0.03) for CON compared to ADY and YEA, while YCA was intermediate. Isobutyrate was greater (*p* = 0.03) in YCA than in CON or ADY with YEA being an intermediate. Valerate was greater (*p* = 0.03) in CON than in the yeast treatments.

### 3.4. Rumen pH and Redox Potential

**Figure 1:**
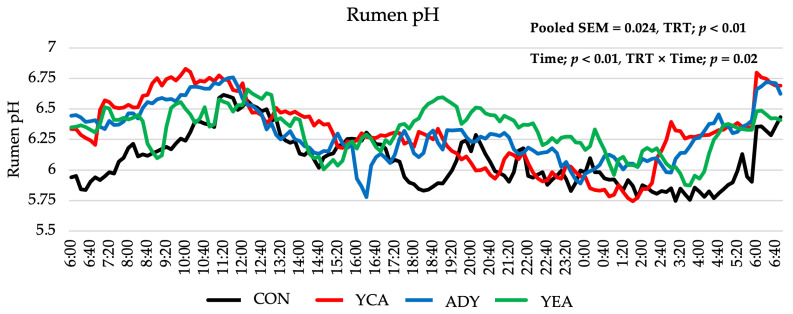
Mean rumen pH was averaged every 10 min for 24 h beginning at 06:00 h, coinciding with milking. Lactating dairy cows were fed a basal control diet with no yeast product (CON) or CON supplemented with 14 g/d yeast culture additive (YCA), 5 g/d active dry yeast (ADY), or 5 g/d active dry yeast plus yeast extract additive (YEA). A treatment × time interaction was observed (*p* = 0.02). Control cows differed from all yeast treatments (*p* < 0.01), whereas yeast treatments did not differ from one another (*p* = 0.52). Rumen pH decreased after feeding (*p* < 0.01), with CON decreasing more than YCA, ADY, and YEA as digestion progressed.

When evaluating treatment effect on ruminal pH, a threshold of 6.0 was used for determining binomial categories. Differences between treatments occurred (χ^2^ = 638.46; *p* < 0.01), CON resulted in greater time in which pH was < 6.0, ADY and YEA resulted in greater time in which pH was > 6.0. Mean rumen pH (Figure 1) was averaged every 10 min over 24 h beginning at 06:00 h, the time of milking, in lactating dairy cows fed different yeast products. A treatment-by-time interaction (*p* = 0.02) occurred. The CON differed from all yeast treatments (*p* < 0.01), with no differences among yeast treatments (*p* > 0.52). Over time, pH decreased after feedings (*p* < 0.01). Rumen pH was the most similar at times of feed consumption, and pH for CON decreased compared to the yeast-fed treatments, YCA, ADY, and YEA as feed digestion occurred over time. These data indicated that ADY and YEA increased the amount of time the rumen environment was maintained at a pH greater than 6.0.

**Figure 2:**
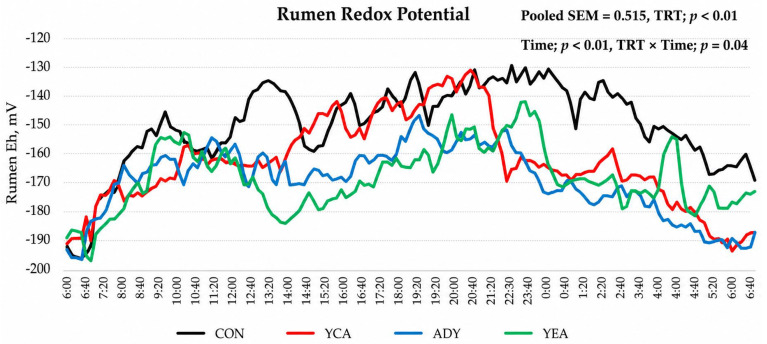
Mean rumen E_h_, an indicator of redox potential, was averaged every 10 min for 24 h using the same sampling period and dietary treatments previously described. A treatment × time interaction was observed (*p* = 0.04). Control cows had greater redox potential than all yeast-supplemented treatments (*p* < 0.01). Among yeast treatments, ADY decreased redox potential compared with YCA (*p* = 0.04), while YEA tended to decrease redox potential compared with YCA (*p* = 0.11). Across time, redox potential increased after feeding (*p* < 0.01).

When evaluating treatment effect on redox potential (Table 4), a threshold of −190 mV was used. Differences between treatments occurred (χ^2^ = 372.25; *p* < 0.01), where YCA had no effect beyond the expected values, CON resulted in greater time in which redox potential was > −190, ADY and YEA resulted in greater time in which redox potential was below −190, with YEA having greater impact than ADY. Mean rumen E_h_ (Figure 2), was averaged every 10 min over 24 h beginning at 06:00 h, the time of milking, in lactating dairy cows fed different yeast products. A treatment-by-time interaction (*p* = 0.04) occurred. The CON treatment increased E_h_ over all yeast treatments (*p* < 0.01). ADY decreased E_h_ over the YCA treatment (*p* = 0.04), while YEA had a tendency to decrease E_h_ over the YCA treatment (*p* = 0.11). Over time, E_h_ increased after feedings (*p* < 0.01). These data indicate that ADY and YEA promoted selected ruminal conditions associated with greater time at pH > 6.0 and lower E_h_, suggesting a more buffered and reducing ruminal environment under the conditions of this study.

## 4. Discussion

Supplementing a yeast product does not impact the lactating dairy cow DMI of a high-concentrate diet due to the nature of yeast’s effectiveness on rumen efficiency [7,22]. Yeast will promote microbial population growth and increase ruminal fiber digestion rate [23]. The purpose of feeding a high-concentrate diet that includes z yeast product, such as YCA, ADY, and YEA, is to increase fiber digestion within the rumen, influencing the rumen environment, with a possibility of impacting DMI intake [24,25]. Decreasing DMI over time was likely attributed to rising ambient temperatures and progression through lactation as the study began with air temperatures at times of less than 0 °C and continued into temperatures of more than 32 °C with increasing humidity and severe fluctuations between treatments. The design of the experiment, 4 × 4 Latin square, removes error from environmental changes with all treatments occurring across the four periods. Cow FCM yield between treatments resulted in no differences, an indication that there were no differences in energy required between treatments to produce the amount of milk measured. Overall, yeast supplementation neither improved nor reduced lactation efficiency under the conditions of this study.

Milk fatty acid profiles can be used to estimate methane production and provide insight into ruminal fermentation patterns when direct methane measurements are not available [15]. Stable rumen fermentation supports fiber digestion and milk fat synthesis, whereas altered ruminal biohydrogenation can contribute to milk fat depression through the production of bioactive fatty acids [26]. Dijkstra et al. [15] developed an equation using specific milk fatty acids to estimate methane output, with C14:0 iso, C15:0 iso, and C17:0 *anteiso* positively associated with methane production and C17:1 and selected biohydrogenation-related fatty acids negatively associated with methane production. In the present study, this approach was used to evaluate potential treatment effects on predicted methane output. Using the Dijkstra et al. [15] equation to estimate methane via FA analysis indicated that treatments did not differ. However, methane production was not measured directly in the present study; therefore, predicted methane output should be interpreted as an estimation rather than a direct measurement. Prediction equations inherently include residual error and may not fully account for animal-level variation, dietary effects, or treatment-specific changes in ruminal fermentation. The lack of treatment differences in predicted methane production should, therefore, be interpreted cautiously; future studies using direct methane measurement techniques are warranted to further evaluate the effects of yeast supplementation on methane production in lactating dairy cows.

The lack of yeast effect on milk SCC, lactose, TS, MUN, MCL, MUFA, and NEFA supports findings by McArt et al. [25] relating negative energy balance to milk components. Milk SCC was not different across treatments. Because it is a cow health and mastitis risk indicator, SCC may have been controlled by the regular cleaning of beds and weekly replacement of sand [27]. Further studies may be done with cows in larger groups and less control of yeast’s effectiveness in preventing high SCC. Fatty acid analysis of MCL, MUFAs, and NEFAs are markers for metabolic disorders and the failure of the cow to transition into the stages of lactation without entering a negative energy balance or SARA. Cows producing MCL over 16 carbons indicate that the cow is at a metabolic disadvantage, FA less than 16 carbons originate in the mammary gland, whereas FA greater than 16 carbons originate from mammary uptake from the plasma. Our results support the claim that the cows observed and sampled were in a constant metabolic state where the rumen environment supported normal milk fat synthesis. The results are also supported by the Souza, Ribeiro and Harvatine [16] finding that carbon chains greater than 16 were non-invasive markers of SARA or altered metabolic state. The lack of NEFAs and MUFAs across treatments indicates that the cows have successfully transitioned into the lactation phase without an adverse effect of decreased DMI or loss of production.

The SNF portion consists of protein (primarily casein and lactalbumin), carbohydrates (primarily lactose), and minerals (including calcium and phosphorus) [28]. Milk SNF tended to increase compared to ADY and YEA. Protein was greater in the CON compared to YCA and YEA, and ADY was not different from any of the treatments. Complete measures of SNF increasing with yeast supplementation indicate a modification to milk production efficiency. Morar et al. [29] likewise reported that supplementation of yeast increased SNF.

The addition of a yeast product increased MF, *de novo* FA as measured in the milk, mixed FA as measured in the milk, and preformed FA as measured in the milk in YCA when compared to CON; however, ADY and YEA were not different from any of the treatments. The effects of YCA may be related to the effect of the specific strain and post-fermentation processing. Relative to total FA, *de novo*, mixed and preformed FA did not differ between treatments. The tendency of increasing MF and *de novo*, mixed and performed FA as a portion of the milk was also reported by Williams et al. [30], when a high-concentrate diet was supplemented with a *S. cerevisiae* product. Yeast effectively increases the FA, *de novo*, mixed, and preformed FA, enhancing production performance [30]. Williams et al. [30] found a diet-by-treatment interaction in increasing milk protein concentrations, an inverse relationship from the results gained in this study, where supplementing a yeast product decreased SNF concentrations. The tendency for increases in MF, FA as measured in milk, mixed FA, and preformed FA shows a positive relationship with yeast supplementation.

The effect of yeast treatments on VFA production resulted in higher pre-prandial and lower post-prandial rumen concentrations. Isobutyrate production contributes to dissolved H_2_ in the rumen; lowering levels of isobutyrate production will decrease the amount of H^+^ available and stabilize pH levels [12,31]. Acetate production for CON cows decreased compared to those fed the yeast treatments. Acetate production increased in relation to lower concentrations of isobutyrate produced and acetate relates to lower dissolved H_2_ levels within the rumen. Increased levels of dissolved H_2_ will inhibit acetate production, thereby decreasing energy metabolism via acetate absorption through the ruminal epithelium [31,32]. Propionate concentrations were greater for CON than YCA and ADY, while YEA maintained intermediate concentrations. In the CON diet, propionate concentrations were higher than acetate concentrations, evidence of greater carbohydrate digestion occurring within the rumen microbial population. The pull from the H_2_ pool produced from acetate using propionate production controls the pH drop associated with high acetate concentrations [32] The acetate:propionate ratio was greater for YCA than CON; ADY and YEA were intermediate and not different than CON or YCA. The acetate:propionate ratio reflects the change in rumen environment as microbes increase fiber digestion [32]. Yeast treatments mitigate the drop in pH brought by consumption of a high NSC diet, as evidenced by decreasing isobutyrate concentrations and increasing acetate:propionate production. Overall, these VFA shifts suggest that yeast supplementation influenced ruminal fermentation patterns under the high-concentrate diet, although responses were limited and should be interpreted alongside pH and redox potential findings.

The rumen is ideally buffered and considered balanced at a pH of 6.8, becoming acidic below a pH of 6.0 with declining fiber digestion. The pH 6.0 threshold indicates cows are at risk of entering SARA. pH probes were placed near the cranial sac of the rumen, a measurement of the lowest pH possible and the best representation of changes in pH over the 24 h measurement period. The chi-squared distribution showed differences among treatments, where YCA had no effect beyond the expected values, CON resulted in greater time in which pH was <6.0, and ADY and YEA resulted in a greater time in which pH was >6.0. Mean rumen pH averaged every 10 min over 24 h show a treatment-by-time interaction. CON differed from all yeast treatments, with no differences among yeast diets. Over time, pH decreased post-prandially. Rumen pH was the most similar at times of feed consumption; pH for CON decreased compared to the yeast-fed treatments, YCA, ADY, and YEA, as feed digestion occurred over time. These data indicated that ADY and YEA increased the time in which the rumen environment maintained a pH > 6.0. However, because dry matter intake, milk yield, production efficiency, and most milk components were not altered, these responses should be interpreted as changes in selected ruminal indicators rather than broad stabilization of rumen function. Therefore, the effects of ADY and YEA are more conservatively described as supporting the ruminal conditions associated with greater time above the pH threshold and lower E_h_ under the conditions of this study. A treatment-by-time interaction indicated yeast supplementation decreased acidosis events driven by high-concentrate diets and support a stable ruminal environment. Pinloche et al. [12] provide support for the interpretation that yeast supplementation can influence ruminal pH dynamics. In that study, yeast supplementation was associated with increased ruminal pH and greater average pH over time, indicating that yeast products may help to buffer ruminal conditions during periods of dietary challenge. These effects may be partly explained by yeast-related support of lactate-utilizing bacteria, oxygen removal, and improved conditions for anaerobic microbial activity. Similarly, in the present study, yeast-supplemented treatments increased the time that ruminal pH remained above the selected threshold, suggesting that yeast supplementation supported selected ruminal conditions associated with pH maintenance. The increase in acetate production and decreases in propionate, butyrate, and valerate production, indicate a positive shift towards a favorable environment for fiber-digesting bacterial populations.

Redox potential allows insight into the specific microbes adapted to specific E_h_ conditions, aerobic bacteria ranging from +500 mV to +300 mV, facultative anaerobes between +300 mV to −100 mV, and anaerobes between +100 to less than −250 mV [19]. E_h_ is the potential difference (E_0_) between a platinum electrode and reference electrode (C) relative to a hydrogen electrode [33]. We used the following equation:

E_h_ = E_0_ + C

The E_0_ must be corrected according to the electrode used relative to the standard hydrogen electrode [34]. After correction, E_h_ values are eligible for comparison across multiple studies using various electrodes, relating the microbial population’s reducing power in the rumen to consistent measurements and removing outliers in raw data [33]. Each microorganism type is adapted to specific E_h_ conditions and is characterized by its ability to develop within a range of E_h_ values. E_h_ is commonly used in a large panel of disciplines dealing with living organisms, as [19] expressed the role of E_h_ to be closely tied to ruminal pH, a major determining factor in biological interactions. Baldwin and Emery [35] concluded that the reductive characteristics of the rumen could be used as an index of fermentation rate in much the same manner as pH. If pH is related to rumen function and E_h_ is indicative of buffering power, then the rumen environment can be characterized using pH and E_h_ as indicators of acetate production, which in turn results in higher MF concentrations.

Redox potential varies depending on time after eating and diet consumed [19]. E_h_ is affected by sample time after feeding, representing oxygen intake upon eating, mastication, and drinking [33]. The lower E_h_ observed with ADY and YEA may be explained by the biological activity of yeast within the rumen. Live yeast products can scavenge oxygen introduced through feed intake, water consumption, rumination, or sampling, thereby supporting the anaerobic conditions required by ruminal microorganisms. A more reducing ruminal environment may favor the fibrolytic bacterial populations involved in fiber degradation and acetate production. Yeast supplementation may also support lactate-utilizing bacteria, reducing lactate accumulation and helping to maintain ruminal pH under high-concentrate dietary conditions. Therefore, the lower E_h_ observed in yeast-supplemented cows may reflect the changes in ruminal redox balance associated with oxygen removal, enhanced anaerobic microbial activity, and the support of fiber-digesting and lactate-utilizing bacterial populations.

The E_h_ value adjustment depends on the type of probe used and the average temperature over the sampling period [34]. The reference electrode used in the HOBO^®^ MX2501 pH and Temperature Data Logger is a 3.5 M potassium chloride electrode (Onset Computer Corporation, Bourne, MA, USA). The average rumen temperature over the 24 h sample period remained at 40 °C, giving a correctional value of −193.3 mV [36]. After correction, E_h_ may be compared across multiple studies using various electrodes, relating the microbial population’s reducing power in the rumen to consistent measurements and removing outliers in raw data [33]. The correctional value allows for accurate comparisons across studies and precise representation of yeast’s interaction in the rumen.

A threshold of −190 mV was used to determine the effectiveness of the yeast’s ability to push the rumen microbes toward fiber digestion [12]. In the present study, ADY and YEA increased the amount of time ruminal E_h_ remained below −190 mV, suggesting that these treatments promoted selected ruminal conditions associated with a more reducing environment. However, because DMI, milk yield, production efficiency, and most milk components were not altered, these findings should be interpreted as changes in selected ruminal indicators rather than broad stabilization of rumen function.

A treatment-by-time interaction reflected a shift in bacterial population toward fiber digestion by decreasing redox potential [12,18]. The production of VFAs by rumen microbial population influences E_h_, with the accumulation of VFAs decreasing pH and increasing redox potential [18,37]. Rumen microbial populations are influenced by the scavenging ability of yeast to acquire oxygen, affecting the H_2_ pool available and increasing pH, thereby decreasing E_h_ [37,38,39]. The role of yeast on E_h_ influences rumen pH, with the availability of H+ ions determined the values reported in this study. [12] examined E_h_ and observed decreasing E_h_ values over time after the application of *S. cerevisiae* Sc47. A decrease in E_h_ indicates the potential oxygen-scavenging activity of the yeast itself and the stimulation in the general bacterial population that is often observed when live yeast is fed to ruminants [12]. Huang et al. [19] supported the conclusion that the shift in rumen environment favors fiber digestion. As E_h_ becomes negative, fiber digestion increases; as redox potential becomes positive, NSC digestion increases. The more negative E_h_ value indicated cellulolytic microbial populations working with the yeast supplement to scavenge O_2_ from the rumen environment [37]. Facultative anaerobic bacteria maintain an anaerobic environment by scavenging oxygen for anaerobic cellulolytic bacteria [32]. Both bacterial populations use O_2_ as a final electron acceptor to drive fermentation reactions and produce VFAs. Knowing that E_h_ relates to pH, with the availability of H^+^ ions being the determining factor in values reported, yeast additives in any diet should influence E_h_. A decrease in E_h_ indicates the potential oxygen-scavenging activity of the yeast itself and the stimulation of population growth of fiber digesting microbial populations that is often observed when live yeast is fed to ruminants [12]. The observed decrease in E_h_ with ADY and YEA may be partially explained by the biological activity of yeast within the rumen. Live yeast products have been reported to scavenge oxygen entering the rumen through feed, water intake, rumination, or sampling, thereby helping maintain the anaerobic conditions required by ruminal microorganisms. A more reducing ruminal environment may favor obligate anaerobic and fibrolytic bacterial populations, which are important for fiber degradation and acetate production. Yeast supplementation may also support lactate-utilizing bacteria, thereby reducing lactate accumulation and helping to maintain ruminal pH under high-concentrate dietary conditions. Therefore, the lower E_h_ observed in yeast-supplemented cows may reflect changes in ruminal redox balance associated with oxygen removal, enhanced anaerobic microbial activity, and support of fiber-digesting and lactate-utilizing bacterial populations.

Rumen E_h_ is negatively correlated with increased pH and acetate concentration resulting in increased fiber digestion [37]. E_h_ is positively correlated with decreases in pH and propionate concentrations resulting in increased NSC digestion. The results show a reductive environment that shifts to fiber digestion and the overall effectiveness of yeast in maintaining the ideal rumen environment for dairy cattle fed a high-concentrate diet due to production pressures.

The results from this study indicate that yeast supplementation impacts VFA production, pH, and redox potential in the diet by favoring microbial populations that digest fiber while improving the rumen environment and mitigating drops in pH related to SARA. However, the method used to collect rumen fluid samples and probe placement may have impacted pH and E_h_ measurements. Cannula use will lead to an increase in dissolved gasses, such as H_2_ and CH_4_ in rumen fluid, skewing measurements from non-cannulated animals [40]. Using a cannula in dairy cows will introduce more oxygen into the rumen environment due to cannula misfits, cap removal, and gaps created between the fistula and cannula when the cows rest.

Future studies may benefit from analyzing pre- and post-prandial pH and E_h_ in relation to VFA production by collection via oral probe or a method that does not expose the rumen environment to oxygen.

## 5. Conclusions

The novelty of the present study was the simultaneous comparison of three different yeast product types, including yeast culture additive (YCA), active dry yeast (ADY), and yeast extract additive (YEA), within the same experimental framework. This comparison allowed for an evaluation of whether different yeast-based additives similarly or differently influenced milk fatty acids, milk components, and ruminal conditions. In the present study, yeast supplementation influenced selected milk components and milk fatty acid responses without increasing dry matter intake, which differs from some previous reports in which yeast products improved intake or production responses. Additionally, treatments containing ADY and YEA increased the frequency that cows maintained a buffered rumen and supported a more reducing ruminal environment, as indicated by greater time above the ruminal pH threshold and lower redox potential.

The predicted methane production associated with yeast extract additives may indicate a shift in the ruminal methanogen population, potentially redirecting metabolic hydrogen toward alternative fermentation end products. However, because methane production was estimated from milk fatty acid profiles rather than measured directly, conclusions regarding methane mitigation should be interpreted conservatively; future studies using direct methane measurement are warranted. This microbial shift may alter rumen fermentation patterns and influence the end products reflected in milk fat synthesis, contributing to improved milk quality. Overall, yeast products appear to have a positive effect on milk components, even as dry matter intake decreases over time, while maintaining consistent levels of milk production.

Yeast supplementation influenced ruminal dynamics, as indicated by changes in acetate production, increased time above the ruminal pH threshold, decreased redox potential, and increased milk fat percentage. These responses suggest that yeast products may support ruminal conditions associated with improved buffering capacity and a more reducing environment under a high-concentrate dietary challenge. Consequently, yeast supplementation may help to moderate selected ruminal responses associated with high-concentrate diets, while supporting milk component responses and maintaining milk production.

## Figures and Tables

**Table 1:** Dietary formulation and nutrient composition of the basal diet fed to mid to late lactating dairy cattle.

Ingredients ^1^, % DM	Value
Corn silage	27.26
Ground corn	23.80
Beet pulp	12.55
Whole cottonseed	10.50
Balage	8.48
SoyPlus^®^	4.91
Canola meal	4.27
Cane molasses	3.16
Vitamin-mineral pre-mix	1.71
Supplement ^2^	1.65
SQ-810™ sodium sesquicarbonate	1.03
Urea	0.49
Salt	0.19
Chemical composition, DM ^3^	
Dry Matter (DM), %	41.35
Crude protein, %	15.98
Acid detergent fiber, %	18.08
Neutral detergent fiber, %	32.03
Starch, %	25.90
Total digestible nutrients, %	71.25
Ash, %	6.70
NE_L_, Mcal/kg	1.67
NE_M_, Mcal/kg	1.56
NE_G_, Mcal/kg	1.00

^1^ Diets were formulated to meet or exceed NASEM (2021) [20] requirements for mid to late lactating dairy cattle. ^2^ Soybean meal-based supplement used to increase trace mineral and amino acid concentration in the diet. ^3^ Values as measured by proximate analysis of pooled feed samples, collected daily, on a dry matter basis except diet DM (Dairy One, Ithaca, NY, USA). Abbreviations: NE_L_, Net energy lactation; NE_M_, Net energy maintenance; NE_G_, Net energy gain.

**Table 2:** Effects of yeast culture additive (YCA), active dry yeast (ADY), and active dry yeast + yeast additive extract (YEA) on fat-corrected milk yield (FCM), dry matter intake (DMI), and milk production efficiency (FCM/DMI) on lactating dairy cows receiving four dietary yeast treatments.

	Treatment ^1^				Pooled SEM ^3^	*p*-Value
Item	CON	YCA	ADY	YEA		
Mean FCM ^2^, kg/d	36.58	39.68	37.82	39.05	1.111	0.88
Mean DMI, kg	23.37	22.99	24.52	23.53	0.333	0.40
Mean Efficiency	1.67	1.68	1.55	1.76	0.061	0.84
CH_4_ Output ^4^, g/kg DMI	20.83	20.05	20.21	19.31	2.083	0.52

^1^ CON (control basal diet) = 0 g/(hd·d) of additional yeast products, CON + 14 g/d of yeast culture additive (YCA), CON + 5 g/d of active dry yeast (ADY), and CON + 5 g/d active dry yeast + yeast extract additive (YEA). ^2^ Fat-corrected milk (FCM) 3.5% = [(0.432 × lb milk) + (16.23 × lb fat)]/2.2 is adjusted according to national Holstein standards in the United States. ^3^ SEM = Standard error of the mean. ^4^ Predicted methane output was calculated using the following equation: CH4 (g/kgDMI) = 24.6 + 8.74 × C17:0 *anteiso* − 1.97 × *trans*-10 + 11C18:1 − 9.09 × *cis-11* C18:1 + 5.07 × *cis-13* C18:1 (individual FA in g/100 g of total FA; R^2^ = 0.73 based on [15].

**Table 3:** Somatic cell count (SCC), milk fat (MF), protein, lactose, solids-not-fat (SNF), total solids (TS), milk urea nitrogen (MUN), *de novo* fatty acids (FA), mixed FA, preformed FA, mean chain length (MCL), mono-unsaturated fatty acids (MUFA), and non-esterified FA (NEFA) content in milk from lactating dairy cows fed a control (CON) diet, 14 g/d of yeast culture additive (YCA), 5 g/d of active dry yeast (ADY), and yeast extract additive (YEA).

	Treatment ^1^					
Component	CON	YCA	ADY	YEA	Pooled SEM	*p*-Value
SCC	250.73	152.78	308.22	109.55	29.945	0.26
MF	3.82 ^y^	4.63 ^x^	4.21 ^xy^	4.44 ^xy^	0.095	0.10
Protein	3.80 ^a^	3.54 ^b^	3.64 ^ab^	3.57 ^b^	0.035	0.01
Lactose	4.51	4.58	4.45	4.55	0.032	0.44
SNF	9.46 ^x^	9.28 ^xy^	9.23 ^y^	9.27 ^y^	0.038	0.12
TS	13.59	14.28	13.81	14.09	0.111	0.16
MUN	12.57	13.12	13.05	13.02	0.172	0.79
*De novo* FA						
g/100 g of milk	0.91 ^y^	1.05 ^x^	0.93 ^xy^	1.03 ^xy^	0.021	0.12
g/100 g of FA	24.89	24.16	23.56	24.55	0.269	0.75
Mixed FA						
g/100 g of milk	1.41 ^y^	1.72 ^x^	1.59 ^xy^	1.71 ^xy^	0.044	0.11
g/100 g of FA	37.97	38.49	39.10	40.19	0.463	0.28
Preformed FA						
g/100 g of milk	1.31 ^y^	1.62 ^x^	1.40 ^xy^	1.42 ^xy^	0.033	0.13
g/100 g of FA	37.14	37.35	37.34	35.26	0.598	0.45
MCL	14.50	14.59	14.62	14.58	0.022	0.60
MUFA	0.19	0.20	0.20	0.19	0.005	0.65
NEFA	669.98	780.09	657.78	662.82	24.188	0.31

^1^ CON (control basal diet) = 0 g/(hd·d) of additional yeast products, CON + 14 g/d of yeast culture additive (YCA), CON + 5 g/d of active dry yeast (ADY), and CON + 5 g/d active dry yeast + yeast extract additive (YEA). ^ab^ Means within same row with different superscripts differ for treatment (*p* ≤ 0.05). ^xy^ Means within same row with different superscripts tend to differ for treatment (*p* ≤ 0.15).

**Table 4:** Ruminal VFA (mmol/100 mL) profile of rumen fluid collected from mid-lactation, ruminally cannulated dairy cows fed different yeast products pre- and post-prandial.

	Treatment ^1^				Pooled SEM	*p*-Value
Ruminal pH *	CON	YCA	ADY	YEA		
Mean pH	6.09	6.28	7.23	6.33	0.000	
Minimum pH	5.07	4.77	5.19	5.13		
Maximum pH	7.50	7.48	7.24	7.15		
pH > 6.0, min/d	3889 ^c^	4583 ^b^	4920 ^a^	4898 ^a^		<0.01
pH < 6.0, min/d	2111 ^a^	1417 ^b^	1080 ^c^	1101 ^c^		<0.01
Ruminal E_h_ **						
Mean E_h_	−149.74	−162.97	−169.39	−168.35	0.010	
Minimum E_h_	−232.95	−221.92	−230.06	−218.50		
Maximum E_h_	−93.22	−70.97	−124.37	−98.31		
E_h_ > 190 mV, min/d	5734 ^a^	5358 ^b^	5196 ^c^	5144 ^c^		<0.01
E_h_ < 190 mV, min/d	266 ^c^	642 ^b^	804 ^a^	856 ^a^		<0.01
Ruminal VFA					Pooled SEM	
Acetate	75.49 ^b^	79.95 ^a^	80.25 ^a^	78.82 ^a^	0.750	0.02
Pre-prandial	76.10	80.23	80.38	78.97		
Post-prandial	74.88	79.67	80.12	78.67		
Propionate	16.00 ^a^	12.72 ^b^	13.25 ^b^	13.97 ^ab^	0.499	0.03
Pre-prandial	15.59	12.40	13.35	13.90		
Post-prandial	16.42	13.03	13.14	14.04		
Butyrate	8.51 ^a^	7.33 ^ab^	6.50 ^b^	7.21 ^b^	0.384	0.03
Pre-prandial	8.31	7.37	6.27	7.13		
Post-prandial	8.70	7.29	6.73	7.28		
Isobutyrate ***	0.71 ^b^	0.90 ^a^	0.72 ^b^	0.85 ^ab^	0.021	0.03
Pre-prandial	0.80	0.94	0.71	0.93		
Post-prandial	0.63	0.86	0.72	0.77		
Valerate	1.75 ^a^	0.74 ^b^	0.78 ^b^	0.76 ^b^	0.133	0.03
Pre-prandial	1.61	0.72	0.74	0.80		
Post-prandial	1.88	0.75	0.83	0.72		
Isovalerate	0.47	0.50	0.44	0.49	0.006	0.42
Pre-prandial	0.49	0.53	0.43	0.51		
Post-prandial	0.46	0.48	0.46	0.47		
Acetate/Propionate	5.56 ^b^	6.42 ^a^	6.12 ^ab^	5.92 ^ab^	0.248	0.03
Pre-prandial	5.62	6.63	6.08	6.01		
Post-prandial	5.50	6.20	6.16	5.82		

^1^ CON (control basal diet) = 0 g/(hd·d) of additional yeast products, CON + 14 g/d of yeast culture additive (YCA), CON + 5 g/d of active dry yeast (ADY), and CON + 5 g/d active dry yeast + yeast extract additive (YEA). ^abc^ Means within same row with different superscripts differ for treatment (*p* < 0.05). * Chi-squared detected differences in the amount of time ruminal pH was above or below pH of 6. ** Chi-squared detected differences in the amount of time ruminal E_h_ was above or below pH of −190 mV. *** Isobutyrate also had a main effect of time where pre-prandial quantities were greater (*p* = 0.05) than post-prandial.

## Data Availability

The datasets generated and analyzed during the current study are not publicly available because they are proprietary and owned by Phileo by Lesaffre. Access to the data is restricted and may only be granted with permission from Phileo by Lesaffre.

## Abbreviations

The following abbreviations are used in this manuscript:

ADY	Active dry yeast
CON	Control
DFM	Direct fed microbial
DM	Dry matter
DMI	Dry matter intake
ECM	Energy corrected milk
E_h_	Redox potential (mV)
FA	Fatty acid
FCM	Fat-corrected milk
H^+^	Hydrogen
H_2_	Dihydrogen
LPS	Lipopolysaccharide
MF	Milk fat
MOS	Mannan-oligosaccharide
MUFA	Mono-unsaturated fatty acid
MUN	Milk urea nitrogen
NEFA	Non-esterified fatty acid
NSC	Non-structural carbohydrate
SARA	Sub-acute ruminal acidosis
SCC	Somatic cell count
SNF	Solids non-fat
TMR	Total mixed ration
TS	Total solids
VFA	Volatile fatty acid
YCA	Yeast culture additive
YEA	Yeast extract additive
